# Beneficial Outcomes of Immunoenhancing Nutritional Interventions in Perioperative Care for Oral Cancer: A Systematic Review and Meta-Analysis

**DOI:** 10.3390/cancers17111855

**Published:** 2025-05-31

**Authors:** Shin-ichiro Hiraoka, Takahiro Abe, Masahiro Watanabe, Daisuke Takeda, Hidemichi Yuasa, Masatoshi Adachi, Narikazu Uzawa, Hiroshi Kurita

**Affiliations:** 1 The Guidelines Committee, Japanese Society of Oral and Maxillofacial Surgeons, Minato-ku, Tokyo 108-0014, Japandsktkd@med.kobe-u.ac.jp (D.T.);; 2 Department of Oral and Maxillofacial Surgery, Graduate School of Dentistry, The University of Osaka, Osaka 565-0871, Japan; 3 Department of Oral and Maxillofacial Surgery, Kanagawa Dental University, Yokosuka 238-8580, Japan; 4 Second Department of Oral and Maxillofacial Surgery, Osaka Dental University, Osaka 565-0871, Japan; 5 Department of Oral and Maxillofacial Surgery, Kobe University Graduate School of Medicine, Kobe 650-0017, Japan; 6 Oral and Maxillofacial Surgery, Toyohashi Medical Center, Toyohashi 440-8510, Japan; 7 Department of Oral and Maxillofacial Surgery, Akiru Municipal Medical Center, Tokyo 197-0834, Japan; 8 Department of Oral & Maxillofacial Oncology and Surgery, Graduate School of Dentistry, The University of Osaka, Osaka 565-0871, Japan; 9 Department of Dentistry and Oral Surgery, School of Medicine, Shinshu University, Matsumoto 390-0802, Japan

**Keywords:** oral neoplasms, perioperative care, immunonutrition, nutritional support, systematic review, meta-analysis

## Abstract

Background: This systematic review aimed to evaluate the efficacy of immunoenhancing nutritional therapy compared to conventional nutritional care in reducing perioperative complications in adult patients undergoing surgery for oral cancer. Given the unclear role of immunonutrition in this specific surgical setting, we synthesized available randomized controlled trials to assess outcomes such as surgical site infections, wound healing complications, hospital stay, and adverse events. Methods: Patients who underwent planned oral cancer surgery were included. The intervention group received oral or enteral immunoenhancing nutritional agents preoperatively, postoperatively, or both, while the control group received standard care (intravenous fluids) and/or macromolecular nutritional supplements. PubMed, Cochrane CENTRAL, and Central Medical Journal were comprehensively searched for randomized controlled trials (RCTs); eight RCTs were included. The primary outcomes were mortality, suture/healing failure, surgical site infection (SSI), and hospital stay length, with evidence certainty assessed using the Grading of Recommendations, Assessment, Development, and Evaluations (GRADE) approach. Results: Although mortality estimation was not feasible, hazard ratios from the meta-analysis showed that the intervention significantly improved suture/healing failure, SSI, and hospital stay length. The certainty of evidence was “low” for suture/healing failure and SSI and “moderate” for hospital stay length. Conclusions: Perioperative management with enteral nutritional agents fortified with immunonutrients should be considered in adult patients scheduled for (advanced) oral cancer surgery.

## Simple Summary

Oral cancer surgery can result in complications and delayed recovery. This study explores whether immunonutritional therapy—nutritional supplements designed to support the immune system—can help reduce such risks compared to standard nutritional care. We reviewed several clinical studies involving adults who received either immune-enhancing supplements or conventional nutrition before, during, or after oral cancer surgery. Our findings suggest that immunonutritional therapy may improve wound healing, reduce surgical site infections, and shorten hospital stays. Although further research is needed to confirm these results, this approach could be a valuable addition to the care of patients undergoing oral cancer surgery.

## 1. Introduction

Oral cancer, a subset of head and neck cancer, remains a significant global health challenge. Surgical intervention is often required, but perioperative complications can negatively affect outcomes. One critical aspect of perioperative care is ensuring optimal nutritional status, which can significantly influence recovery and complication rates [1]. Previous research has demonstrated that nutritional interventions, particularly those that modulate immune function, may reduce postoperative complications in various surgical fields [2,3]. Immunoenhancing nutritional agents—such as arginine, omega-3 fatty acids, and glutamine—have been shown to support immune responses, reduce inflammation, and promote wound healing [4,5]. These agents are collectively referred to as immunonutrition. While immunonutrition has shown beneficial effects in gastrointestinal and other surgical fields, its specific application in oral cancer surgery has not been thoroughly investigated. A previous systematic review on head and neck cancer patients [6] included some oral cancer cases but did not provide focused analysis or subgroup evaluation. Moreover, the evidence was drawn from heterogeneous populations and interventions. Therefore, our review aims to clarify whether immunoenhancing nutritional agents provide perioperative benefits specifically in oral cancer surgery, a population with unique anatomical and nutritional challenges.

However, despite promising results observed in various surgical disciplines, the specific role of immunoenhancing nutritional interventions in the perioperative care of patients with oral cancer remains uncertain [7]. While some previous studies have suggested a potential benefit in terms of reduced postoperative complications and shorter hospital stays [8,9], others have reported mixed results. This inconsistency necessitates a more comprehensive understanding of this topic, especially given the unique challenges associated with oral cancer surgery, such as the potential for postoperative impaired oral intake.

This systematic review (SR) was conducted to collate and analyze the available evidence on the efficacy of immunoenhancing nutritional interventions in the perioperative care of patients with oral cancer. By comparing outcomes between patients receiving immunonutrition and those receiving conventional nutritional therapy, we aim to clarify its clinical value and inform perioperative management strategies in oral oncology.

## 2. Materials and Methods

### 2.1. Study Details

This systematic review and meta-analysis was conducted following the PRISMA 2020 guidelines [10,11] (PRISMA-2020; (Supplementary Materials Table S1)). The protocol was registered with PROSPERO (CRD 42024558163).

The results are expressed as risk ratios or mean differences. Data synthesis was performed using Review Manager (RevMan) Version 5.4 (The Cochrane Collaboration).

### 2.2. Search Strategy and Database Selection

We conducted a comprehensive literature search across three major databases: PubMed, Cochrane Central Register of Controlled Trials (CENTRAL), and Ichushi-Web (a Japanese medical database). These databases were selected to ensure coverage of both international and Japanese clinical research, considering that a significant number of relevant studies in oral cancer are published in Japan. Although databases such as SCOPUS, EMBASE, and LILACS were not included in this review, we acknowledge this as a limitation and address it in the Discussion. While no language restrictions were applied during the search, we also recognize the potential for publication bias due to the exclusion of certain grey literature and non-indexed sources. The search period ranged from 15 February 2018 to 28 March 2022. This time frame was chosen to supplement the existing Cochrane systematic review [6], which covered literature up to early 2018. Our study thus aims to provide an updated and focused synthesis on oral cancer, expanding upon the previous broader head and neck cancer reviews. The detailed search strategy is provided in Supplementary Table S2.

### 2.3. Study Selection Criteria and Literature Search

Two authors (SH and MW) independently screened all identified studies in a two-phase process. Titles and abstracts were first screened based on eligibility, followed by full-text review. Disagreements were resolved through discussion with a third reviewer (TA or DT).

Eligibility was assessed using the PICO framework, as detailed below:

Population (P): Adult patients (≥18 years) undergoing surgery for oral cancer.

Intervention (I): Perioperative immunonutrition administered either preoperatively, postoperatively, or both. Immunonutrition included formulas containing arginine, glutamine, omega-3 fatty acids, and/or nucleotides.

Comparison (C): Standard nutritional care (e.g., intravenous fluids or polymeric enteral formulas without immunoenhancing components).

Outcomes (O): Studies were included if they reported at least one of the following:

- Surgical site infection (primary outcome);
- Suture or healing complications;
- Length of hospital stay;
- Adverse events related to nutrition;
- Mortality (within 30 days).

Studies with missing or unclear data were resolved by contacting the original authors.

### 2.4. Inclusion and Exclusion Criteria

Inclusion criteria:

1. Study design: Randomized controlled trials (RCTs).
2. Participants: Adults aged 18 years and older.
3. Population: Studies in which ≥20% of participants had oral cancer, or subgroup data specific to oral cancer could be extracted.
4. Intervention: Immunonutrition administered orally or enterally in the perioperative period (pre-, post-, or both). Immunonutrition was defined as formulas containing immune-modulating components such as arginine, glutamine, omega-3 fatty acids, and nucleotides.
5. Comparator: Standard nutritional care (e.g., IV fluids or non-immunoenhanced polymeric formulas).
6. Reported at least one outcome of interest (see Section 2.5).

Exclusion criteria:

- Non-randomized or quasi-randomized studies;
- Studies involving patients under 18 years;
- Head and neck cancer studies lacking oral cancer-specific data;
- Interventions not meeting the definition of immunonutrition;
- Duplicated reports, abstracts only, or protocol-only studies.

Oral cancer was defined by ICD-10 anatomical categories: oral tongue, floor of mouth, buccal mucosa, hard palate, upper and lower gingiva, and retromolar trigone.

### 2.5. Study Selection Process

Eligible studies were selected in two phases. In the first phase, two authors (SH and MW) independently screened the titles and abstracts. In the second phase, the same authors independently reviewed the full texts of potentially eligible studies based on the inclusion and exclusion criteria described in Section 2.4. Irrelevant studies were excluded. Disagreements were resolved through discussion with a third author (TA or DT). In cases of missing or unclear data, the original study authors were contacted for clarification.

### 2.6. Quality Assessment of Included Studies

The risk of bias for the included RCTs was evaluated using the Cochrane Risk of Bias 2.0 (RoB 2) tool [12,13]. The following domains were assessed:

1. Randomization process;
2. Deviations from intended interventions;
3. Missing outcome data;
4. Measurement of outcomes;
5. Selection of reported results.

Each domain and the overall risk were rated as low risk, high risk, or unclear. Two independent reviewers (SH and MW) conducted the assessments, with discrepancies resolved by discussion.

### 2.7. Summary of Findings

A “Summary of Findings” table (Table 1) was created to compare immunonutritional therapy with standard nutritional care for each outcome. The GRADE (Grading of Recommendations, Assessment, Development, and Evaluations) approach was used to assess the overall certainty of evidence, which was rated as very low, low, moderate, or high [14]. This helps in interpreting the reliability and clinical applicability of the results.

### 2.8. Statistical Analysis and Data Synthesis

Meta-analyses were conducted to compare immunonutritional therapy with standard nutritional care. Hazard ratios (HRs) and 95% confidence intervals (CIs) were calculated for all relevant outcomes. A fixed-effects model was applied using Review Manager (RevMan) Version 5.4 (The Cochrane Collaboration). Heterogeneity was assessed using chi-square tests and the **I^2^ statistic. Values of I^2^ < 40% were considered to indicate low or no heterogeneity, 50–75% mild heterogeneity, and >75% substantial heterogeneity.

## 3. Results

### 3.1. Literature Search and Selection

**Figure 1:**
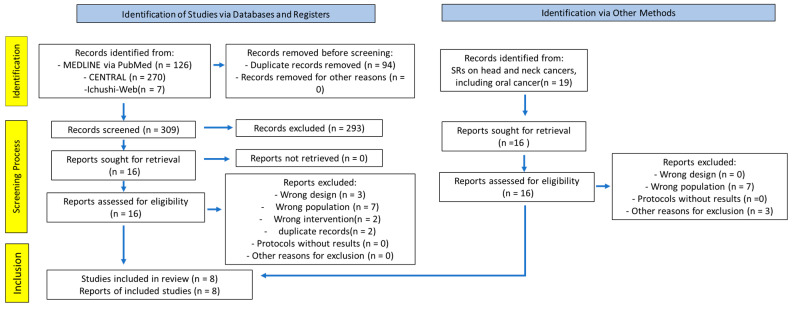
PRISMA 2020 flow diagram of this study.

Figure 1 illustrates the PRISMA flow diagram. SRs on head and neck cancers, including oral cancer, were identified as key SRs, and 16 RCTs obtained from these SRs were included [6]. The combined search of Cochrane CENTRAL, PubMed, and Ichushi-Web yielded 309 articles (PubMed, 126; CENTRAL, 270; Ichushi-Web, 7; duplicates, 94). All titles and abstracts were screened, and 293 articles were excluded. Subsequently, 32 studies were eligible for full-text screening. After full-text screening, 24 articles were excluded primarily due to incompatible study designs and domains (Supplementary Materials Table S3). We reviewed the criteria and outcomes of each study, as well as the references. Eight articles were finally included in the analysis [15,16,17,18,19,20,21,22]. 

### 3.2. Data Extraction

The eight identified RCTs compared perioperative immunonutrition with standard nutritional therapy in adult patients undergoing scheduled surgery for oral cancer. The outcomes and corresponding numbers of eligible patients were as follows: suture and healing failure, 493 (eight RCTs) patients; SSI, 513 (eight RCTs) patients; length of hospital stay, 539 (seven RCTs) patients; and adverse events related to immunonutrition, 369 (five RCTs) patients. Table 1 summarizes the findings and presents the key characteristics and results of the included studies. Table 2 provides a summary of the included RCTs, outlining their characteristics, timing of intervention, reported outcomes, and notable findings.

**Table 1:** Summary of findings.

Certainty Assessment						Sample size		Effectiveness		Certainty of the Evidence (Grade)
**No. of Studies**	**Study Design**	**Risk of Bias**	**Inconsistency**	Non-Directivity	Imprecision	Immunonutrition	Standard	Relative Effect	Absolute Effect	
								(95% CI)	(95% CI)	
**Surgical site infection**										
8	RCT	Serious ^a^	Not serious	Not serious	Serious ^b^	21/265 (7.9%)	32/248 (12.9%)	RR 0.61	50 fewer per 1000	⊕⊕⊝⊝
								(0.37–1.01)	(81 fewer to 1 more)	Low
							10.00%		39 fewer per 1000	
									(63 fewer to 1 more)	
							40.00%		156 fewer per 1000	
									(252 fewer to 4 more)	
**Incidence of suture and healing anomalies**										
8	RCT	Serious ^a^	Not serious	Serious ^d^	Not serious	19/257 (7.4%)	41/236 (17.4%)	RR 0.46	94 fewer per 1000	⊕⊕⊝⊝
								(0.27–0.80)	(127 fewer to 35 fewer)	Low
**Nutritional adverse events**										
5	RCT	Not serious	Not serious	Not serious	Serious ^b^	42/192 (21.9%)	27/177 (15.3%)	RR 1.47	72 more per 1000	⊕⊕⊕⊝
								(0.87–2.48)	(20 fewer to 226 more)	Moderate
**Number of hospital days**										
7	RCT	Serious ^a^	Not serious	Not serious	Not serious	294	245	-	Median < 3.06	⊕⊕⊕⊝
										Moderate
**Mortality**										
^a, b, c^								Not estimable		-

CI, confidence interval; MD, mean difference; RR, risk ratio. Description: ^a^ Domain 4 had a larger number of high-risk studies, and the studies as a whole had a larger number of high-risk studies. ^b^ Because the 95% CI spans from no effect to trivial effect. ^c^ Because the 95% CI straddles no effect to trivial undesirable effect. ^d^ Because most participants were females in the de Luis et al. study [16,17,18], which accounted for approximately 28% of the meta-analysis. Symbols indicate the certainty of evidence according to the GRADE approach: ⊕⊕⊕⊕ High certainty; ⊕⊕⊕⊝ Moderate certainty; ⊕⊕⊝⊝ Low certainty; ⊕⊝⊝⊝ Very low certainty.

### 3.3. Outcome Evaluation of Meta-Analysis

We analyzed five key outcomes across the included randomized controlled trials (RCTs). A summary of the findings is provided below, organized by outcome type. The GRADE approach was used to assess the certainty of evidence.

**▶ Surgical Site Infection (SSI)**

Patients included: 513 across eight RCTs.

Effect estimate: RR = 0.61; 95% CI: 0.37 to 1.01.

Certainty: low.

**Figure 2:**
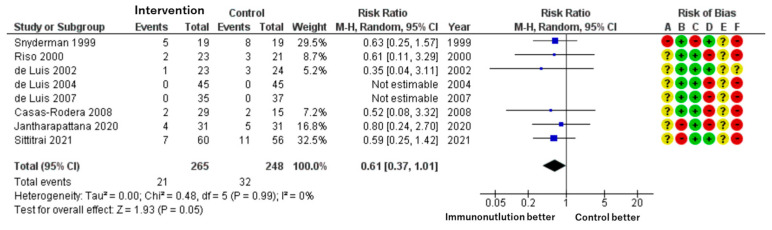
Forest plot of surgical site infection (SSI) and risk of bias across studies.

Immunonutrition was associated with a lower risk of SSI compared to standard care. The absolute reduction was approximately 50 fewer infections per 1000 patients. However, the confidence interval includes the possibility of no effect. The baseline risk in control groups ranged from 3% to 41%, as reported by Cannon et al. [23] (Figure 2).

**▶ Suture or Healing Failure**

Patients included: 493 across eight RCTs.

Effect estimate: RR = 0.46; 95% CI: 0.27 to 0.80.

Certainty: low.

**Figure 3:**
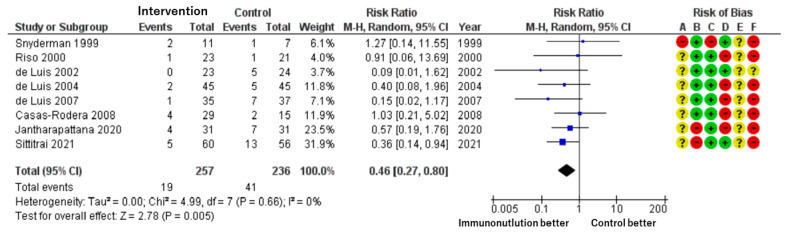
Forest plot of suture or healing failure (e.g., fistula formation) and risk of bias.

A significantly lower rate of suture or wound healing complications (e.g., fistula formation) was observed in the immunonutrition group. The incidence was reduced by 94 per 1000 patients, from 17.4% in the control group to 7.4% in the intervention group (Figure 3).

**▶ Adverse Events Related to Nutritional Intervention**

Patients included: 369 across five RCTs.

Effect estimate: RR = 1.47; 95% CI: 0.87 to 2.48.

Certainty: moderate.

**Figure 4:**
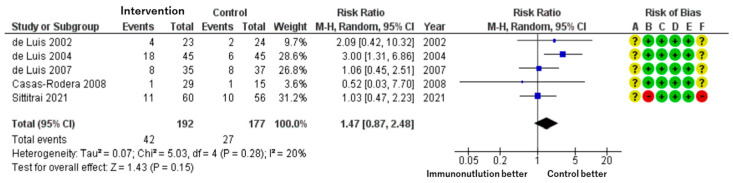
Forest plot of adverse events related to immunonutrition and associated risk of bias.

Although adverse events such as nausea, diarrhea, and abdominal discomfort were more frequently reported in the immunonutrition group, the difference was not statistically significant. The estimate suggests a possible 72 more events per 1000 patients, but the wide confidence interval indicates imprecision (Figure 4).

**▶ Length of Hospital Stay**

Patients included: 539 across seven RCTs.

Effect estimate: mean difference = −3.06 days; 95% CI: −5.42 to −0.70.

Certainty: moderate.

**Figure 5:**
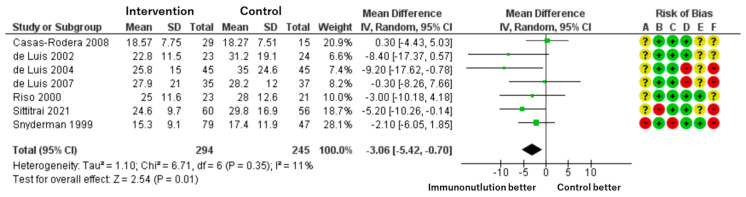
Forest plot of length of hospital stay and corresponding risk of bias.

Immunonutrition shortened the length of hospital stay by an average of 3 days compared to standard nutrition. The effect size was statistically significant and consistent across studies, though some risk of bias remains (Figure 5).

**▶ Mortality**

Data on mortality were insufficient for meta-analysis. None of the included studies reported mortality as a primary endpoint, and data were inconsistently presented across trials. Therefore, no pooled estimate was calculated.

### 3.4. Risk of Bias in Included Studies

We assessed each included study for risk of bias using the Cochrane Risk of Bias assessment tool [24] with the five domains and overall bias, as described in the methods section. Among the identified studies, overall bias showed some concerns related to the whole outcome, and there was at least one concern in several domains (Figure 2, Figure 3, Figure 4 and Figure 5).

### 3.5. Certainty of Cumulative Evidence

The certainty of the evidence for each outcome was assessed using the GRADE approach (Table 1).

For the outcomes suture/healing failure and SSI, the evidence was of low certainty owing to the serious risk of bias in several domains, imprecision of effect estimates (wide CIs), and indirectness (one study with a predominantly female population).

The evidence for the outcomes adverse events related to nutritional supplements and length of hospital stay was of moderate certainty. There was serious imprecision in the effect estimate for adverse events, but no other factors affected the certainty. For the length of hospital stay, there was a serious risk of bias in several domains, but no other factors affected the certainty.

Overall, the certainty of the evidence ranged from low to moderate, suggesting that further research might have impacted the confidence in the effect estimates and may, therefore, change the estimates. The outcomes mortality, suture/healing failure, and SSI were considered critical for decision-making, whereas adverse events and length of hospital stay were considered important.

There were insufficient studies to assess publication bias using funnel plots since the number of included studies for each outcome was below 10.

In conclusion, while the evidence suggests the potential benefits of perioperative immunonutrition in reducing suture/healing failure, SSI, and length of hospital stay, the certainty of the evidence was limited by the risk of bias, imprecision, and indirectness. More high-quality RCTs remain warranted to improve the level of confidence in the effect estimates and assess the impact on mortality.

## 4. Discussion

This SR and meta-analysis comprehensively evaluated the potential benefits of immunonutrition in the perioperative management of patients undergoing surgery for oral cancer. The results suggest that administering immunonutrients, such as arginine, omega-3 fatty acids, and nucleotides, significantly affects surgical outcomes, reducing the incidence of complications, such as suture and healing failure and SSIs, while facilitating faster recovery and shorter hospital stays. Although the meta-analysis demonstrated a statistically significant reduction in hospital stay, the relatively wide confidence interval (−5.42 to −0.70 days) suggests variability across studies and limits the precision of this finding. This indicates that while immunonutrition may reduce hospital stay, the exact magnitude of benefit remains uncertain and should be interpreted with caution. These findings are consistent with the evidence supporting the use of immunonutrition in various surgical contexts, particularly in gastrointestinal surgery [25,26].

In gastrointestinal surgery, numerous studies have demonstrated the efficacy of immunonutrition in reducing postoperative complications and improving clinical outcomes. In a meta-analysis, Drover et al. [27] found that immunonutrition in patients undergoing elective gastrointestinal surgery significantly reduced infectious complications and length of hospital stay compared with standard nutrition. Similarly, an SR by Marimuthu et al. [28] reported that immunonutrition was associated with a lower incidence of wound infections and shorter hospital stays in patients undergoing surgery for gastrointestinal cancers.

The consistency of these findings across different surgical specialties underscores the potential of immunonutrition as a valuable adjunct in perioperative care. However, it is essential to note that the evidence supporting immunonutrition in patients undergoing oral cancer surgery is less robust than that in those undergoing gastrointestinal surgery. This disparity highlights the need for further research to consolidate the findings of the present meta-analysis and explore the specific nuances of immunonutrition in the context of oral cancer.

Although anatomical and functional differences exist between oral cancer and gastrointestinal surgeries, the consistent benefits of immunonutrition observed in various surgical domains suggest its potential applicability in oral oncology. Our findings offer preliminary support for this concept.

One key challenge in the perioperative management of patients with head and neck cancer is the high prevalence of malnutrition, which can reach 50% in this population [29]. Malnutrition compromises immune function, impairs wound healing, and increases the risk of postoperative complications [30]. Therefore, incorporating immunonutrition into perioperative care protocols may help address the unique nutritional challenges faced by patients with oral cancer and improve surgical outcomes.

However, successful implementation of immunonutrition in the case of oral cancer surgery requires a multidisciplinary approach that considers the specific needs and challenges faced by this patient population. For instance, anatomical and functional alterations resulting from oral cancer surgery may necessitate the use of alternative feeding routes, such as enteral tube feeding or parenteral nutrition [31].

Integrating immunonutrition into these feeding strategies requires careful planning and coordination among healthcare teams to ensure optimal delivery and patient compliance [32].

Moreover, the potential economic implications of incorporating immunonutrition into perioperative care protocols must be considered. The possibly higher upfront costs of immunonutrition formulas compared to those of standard nutrition will be compensated for by the potential cost savings associated with reduced complications and shorter hospital stays [33]. Future studies should include robust cost-effectiveness analyses to provide a more comprehensive understanding of the financial impact of immunonutrition in the perioperative care of oral cancer patients.

Further high-quality, large-scale randomized controlled trials (RCTs) are needed to validate the effectiveness of immunonutrition in patients undergoing oral cancer surgery. Future studies should employ well-defined inclusion criteria, clearly delineate primary outcomes such as surgical site infection or healing failure, and explore long-term endpoints such as survival, quality of life, and cost-effectiveness. Additionally, protocol standardization across institutions and inclusion of diverse populations will be critical to strengthen the generalizability and clinical impact of future findings.

## 5. Limitations

This systematic review has several limitations. First, although the number of randomized controlled trials focusing specifically on oral cancer was limited, we included studies where oral cancer patients constituted ≥20% of the population or could be extracted through subgroup analysis. As such, some clinical heterogeneity remains. Second, while no language restrictions were applied and Japanese literature was included via Ichushi-Web, the search did not encompass databases such as SCOPUS, EMBASE, LILACS, or grey literature, which may have led to publication bias. Third, our search period was set from 2018 to 2022, not to merely update or supplement a previous Cochrane review, but because that review—although comprehensive—focused on head and neck cancers broadly and did not specifically target oral cancer. However, since it included relevant trials involving oral cancer patients, we considered it a key systematic review and used it to define the starting point for our literature search. Lastly, one of the included studies had a predominantly female sample, which may limit the generalizability of some findings.

## 6. Conclusions

The results of this SR and meta-analysis provide compelling evidence of the potential benefits of immunonutrition in the perioperative management of patients with oral cancer. Although the evidence base may not be as extensive as that for gastrointestinal surgery, the consistency of findings across different surgical specialties underscores immunonutrition as a valuable adjunct to perioperative care. Moving forward, it is crucial to refine protocols, elucidate the underlying mechanisms, and optimize the delivery of immunonutrition in the context of oral cancer surgery. By fostering interdisciplinary collaboration, promoting patient-centered care, and leveraging lessons learned from other surgical specialties, we can unlock the full potential of immunonutrition and revolutionize the perioperative management of oral cancer, ultimately improving the lives of patients.

## Figures and Tables

**Table 2:** Summary of included studies and key findings: characteristics of the included randomized controlled trials (RCTs), including country, sample size, timing of immunonutrition intervention, primary outcomes assessed, and notable findings. This table provides a concise overview to facilitate comparison across studies.

Study (Author, Year)	Country	Sample Size (*n*)	Intervention Timing	Key Outcomes Reported	Notable Findings
Casas-Rodera et al., 2008 [15]	Spain	40	Postoperative	SSI, Healing Failure	Reduced SSI and fistula rates
de Luis et al., 2002 [16]	Spain	34	Postoperative	Healing Failure, Adverse Events	Improved healing; mild gastrointestinal side effects
de Luis et al., 2004 [17]	Spain	38	Postoperative	SSI, Hospital Stay	Shorter hospital stay; fewer infections
Riso et al., 2000 [19]	Italy	60	Postoperative	Healing Failure, Adverse Events	Reduced wound complications
Snyderman et al., 1999 [20]	USA	36	Perioperative	SSI, Healing Failure	Significant SSI reduction
Jantharapattana et al., 2020 [21]	Thailand	50	Preoperative	Hospital Stay, Nutritional Status	Improved preoperative weight maintenance
Sittitrai et al., 2021 [22]	Thailand	98	Perioperative	SSI, Healing Failure	Moderate reduction in complications
de Luis et al., 2007 [18]	Spain	36	Postoperative	Adverse Events	Higher gastrointestinal side effects in intervention group

## Data Availability

The data supporting this systematic review and meta-analysis are available from the corresponding author upon reasonable request.

## Supplementary Materials

The following supporting information can be downloaded at https://www.mdpi.com/article/10.3390/cancers17111855/s1: Table S1: PRISMA 2020 Checklist; Table S2: The detailed search strategy; Table S3: Characteristics of studies excluded from the qualitative and quantitative synthesis. Reference [34] is cited in the supplementary materials.
