# Supplementary material for: Beneficial Outcomes of Immunoenhancing Nutritional Interventions in Perioperative Care for Oral Cancer: A Systematic Review and Meta-Analysis
Source: Cancers (Basel). 2025 May 31;17(11):1855. doi: 10.3390/cancers17111855 (PMC12153621; doi:10.3390/cancers17111855)
Supplement: Supplementary file 1 [file cancers-17-01855-s001.zip › Table S2.pdf]

Table S2.

①PubMed（MEDLINE）

("Head and Neck Neoplasms"[MeSH Terms] OR (("intraoral*"[Title/Abstract] OR "oral*"[Title/Abstract] OR "mouth*"[Title/Abstract] OR "gingiva*"[Title/Abstract] OR "head and neck"[Title/Abstract] OR "tongue"[Title/Abstract] OR "palatal"[Title/Abstract]) AND ("malignant"[Title/Abstract] OR "carcinom*"[Title/Abstract] OR "neoplas*"[Title/Abstract] OR "cancer"[Title/Abstract] OR "tumor"[Title/Abstract]))) AND ("surgical procedures, operative"[MeSH Terms] OR ("surg*"[Title/Abstract] OR "excis*"[Title/Abstract] OR "resect*"[Title/Abstract] OR "dissect*"[Title/Abstract] OR "exeresis"[Title/Abstract] OR "remov*"[Title/Abstract] OR "operat*"[Title/Abstract])) AND ("Nutrition Therapy"[MeSH Terms] OR "diet, food, and nutrition"[MeSH Terms] OR ("Nutritional Support"[Title/Abstract] OR "Enteral Nutrition"[Title/Abstract] OR "Parenteral Nutrition"[Title/Abstract] OR "Enteral Feeding"[Title/Abstract] OR "Parenteral Feeding"[Title/Abstract] OR "nutritional supplementation"[Title/Abstract] OR "artifical nutrition"[Title/Abstract]) OR "Immunonutrition"[Title/Abstract]) AND (("randomized controlled trial"[Publication Type] OR "controlled clinical trial"[Publication Type] OR "randomized"[Title/Abstract] OR "placebo"[Title/Abstract] OR "clinical trials as topic"[MeSH Terms:noexp] OR "randomly"[Title/Abstract] OR "trial"[Title]) NOT ("animals"[MeSH Terms] NOT "humans"[MeSH Terms])) Filters: from 2018/2/15 - 2022/3/28

②CENTRAL

#1	MeSH descriptor: [Head and Neck Neoplasms] explode all trees
#2	intraoral*:ti,ab
#3	oral*:ti,ab
#4	mouth*:ti,ab
#5	gingiva*:ti,ab
#6	head and neck:ti,ab
#7	tongue:ti,ab
#8	palatal:ti,ab
#9	#2 or #3 or #4 or #5 or #6 or #7 or #8
#10	malignant:ti,ab
#11	carcinom*:ti,ab
#12	neoplas*:ti,ab

#13 cancer:ti,ab
#14 tumor:ti,ab
#15 #10 or #11 or #12 or #13 or #14
#16 #9 and #15
#17 #1 or #16
#18 MeSH descriptor: [Surgical Procedures, Operative] explode all trees
#19 surg*:ti,ab
#20 excis*:ti,ab
#21 resect*:ti,ab
#22 dissect*:ti,ab
#23 exeresis:ti,ab
#24 remov*:ti,ab
#25 operat*:ti,ab
#26 #19 or #20 or #21 or #22 or #23 or #24 or #25
#27 #18 or #26
#28 #17 and #27
#29 MeSH descriptor: [Nutrition Therapy] explode all trees
#30 MeSH descriptor: [Diet, Food, and Nutrition] explode all trees
#31 Nutritional Support:ti,ab 4419
#32 Enteral Nutrition:ti,ab 4929
#33 Parenteral Nutrition:ti,ab 4913
#34 Enteral Feeding:ti,ab 3170
#35 Parenteral Feeding:ti,ab 1181
#36 nutritional supplementation:ti,ab
#37 artifical nutrition:ti,ab 4
#38 #31 or #32 or #33 or #34 or #35 or #36 or #37
#39 Immunonutrition:ti,ab 364
#40 #29 OR #30 OR #38 OR #39 86875
#41 #28 and #40 with Cochrane Library publication date Between Feb 2018 and Mar 2022

③Ichushi-web(in Japanese)

(((頭頸部腫瘍/TH) or (((口腔/TA) or (歯肉/TA) or (舌/TA) or (口蓋/TA) or (口底/TA) or (頬粘膜/TA)) and ((癌/TA) or (悪性/TA))) and ((外科手術/TH) or (切除/TH)) and ((栄

養療法/TA) or (経腸栄養/TA) or (静脈栄養/TA) or (栄養補給/TA) or (栄養補助/TA) or (微量栄養素/TA) or (中心静脈栄養/TA) or (消化態栄養剤/TA) or (半消化態栄養剤/TA) or (経腸成分栄養剤/TA) or (経腸栄養剤/TA) or (栄養機能食品/TA) or (栄養サポートチーム/TA) or (高カロリー輸液用基本液-アミノ酸液/TA) or (免疫アジュバンド/TA)))) and ((PT=症例報告・事例除く) and (PT=会議録除く) and RD=ランダム化比較試験,準ランダム化比較試験,比較研究) and (PDAT=2018/2/15:2022/3/28)
