# Supplementary material for: Beneficial Outcomes of Immunoenhancing Nutritional Interventions in Perioperative Care for Oral Cancer: A Systematic Review and Meta-Analysis
Source: Cancers (Basel). 2025 May 31;17(11):1855. doi: 10.3390/cancers17111855 (PMC12153621; doi:10.3390/cancers17111855)
Supplement: Supplementary file 1 [file cancers-17-01855-s001.zip › Table S3.pdf]

| Table S3. Characteristics of studies excluded from the qualitative and quantitative synthesis | |
|---|---|
| Study | Reason for exclusion |
| Hanai N , et al. Japanese journal of clinical oncology 2018; 356-361. | Wrong population |
| Basmisirli, E, et al.Clinical nutrition ESPEN, 2021, 46, S750. | Wrong population |
| Aeberhard, C , et al. 2017; nan. | Wrong study design |
| Palma-Milla, S, et al. Journal of parenteral and enteral nutrition, 2018, 42(2), 371 - 379. | Wrong population |
| Lages, PC, et al. British Journal of Nutrition (2018), 119, 190–195. | Wrong intervention |
| NCT03531190,Cochrane Central Register of Controlled Trials (CENTRAL).30 June 2018 | Wrong intervention |
| Azman, M, et al. Clinical Nutrition 38 (2019) S59 | Wrong study design |
| Odgaard, KM, et al. Clinical Nutrition 38 (2019) S59. | Wrong population |
| DRKS00016020, nan 2018; nan. | duplicate records |
| Ghosh, S, et al.e-SPEN Journal 2012; 7(3) e107 | Wrong population |
| Hanai N, et al. Japanese Journal of Clinical Oncology 2018; 356-361. | duplicate records |
| Di Renzo L, et al. European review for medical and pharmacological sciences 2019; 1322-1334. | Wrong population |
| de Carvalho CS, et al. Parenteral and Enteral Nutrition 2021; 665-672. | Wrong population |
| RBR-25zmjm, CENTRAL: 31 2019 Issue 3 | Wrong study design |
| D A de Luis , et al.Eur J Clin Nutr 2003; 96-99. | Wrong population |
| D A de Luis, et al. Eur J Clin Nutr 2005; 145-147. | Wrong population |

| | |
|---|---|
| D A De Luis, et al. Eur Rev Med Pharmacol Sci.　2009; 279-283. | Wrong population |
| Daniel de Luis, et al. Nutr Hosp 2014; 870-875. | Wrong population |
| M N Falewee, et al. Clin Nutr 2014; 776-784. | Wrong population |
| Felekis DE, et al.l Nutrition. 2005; 296. | others |
| Dimitrios F, et al. Nutr Cancer 2010; 1105-1112. | others |
| Douglas S, et al. Laryngoscope 2009; 1358-1364. | Wrong population |
| Amy T, et al. Nutrients.　2013; 1186-1199. | others |
| M A Van Bokhorst, et al. Clin Nutr 2000; 437-444. | Wrong population |
